# Supplementary material for: Splice-Junction-Based Mapping of Alternative Isoforms in the Human Proteome
Source: Cell Rep. Author manuscript; Available in PMC 2020 Jan 15. (PMC6961840; doi:10.1016/j.celrep.2019.11.026)
Supplement: 2 [file NIHMS1546469-supplement-2.zip › DF1/PXD009737/TestisTrypsin/TestisTrypsin_15_SMARCC1_PGDVQGPGTAVGATGSGIAAAAAGLAVYR.pdf]

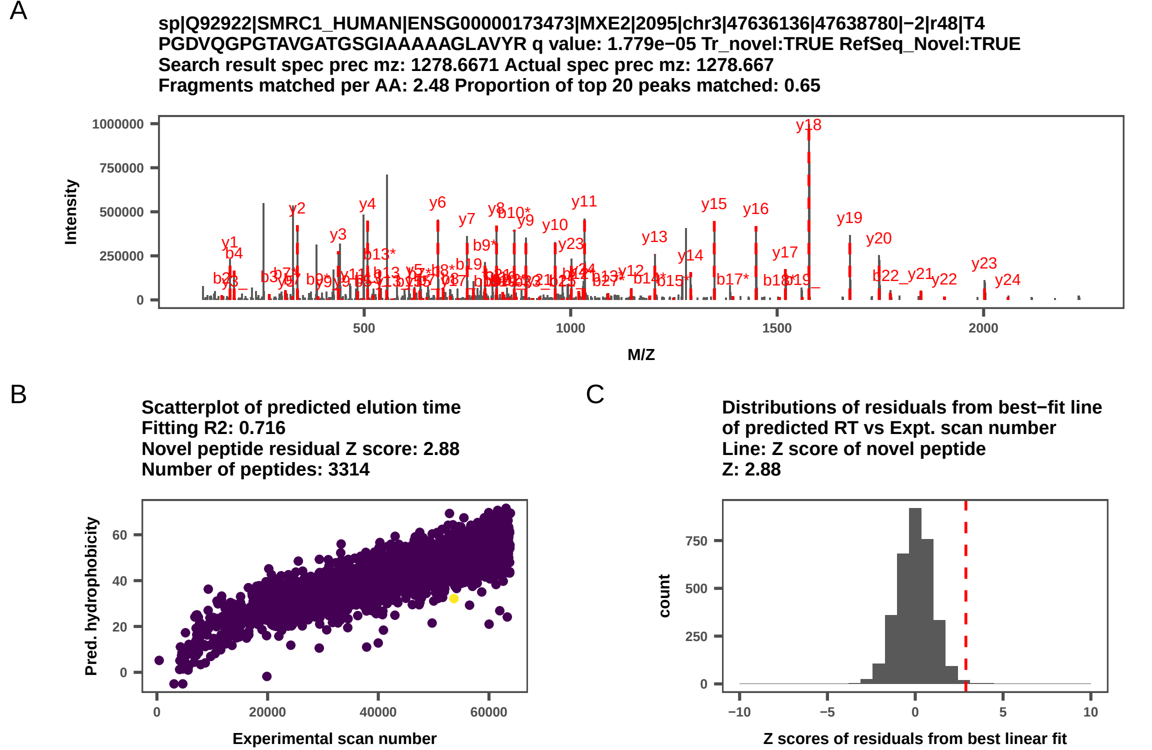
A
sp|Q92922|SMRC1_HUMAN|ENSG00000173473|MXE2|2095|chr3|47636136|47638780|−2|r48|T4
PGDVQGPGTAVGATGSGIAAAAAGLAVYR q value: 1.779e−05 Tr_novel:TRUE RefSeq_Novel:TRUE
Search result spec prec mz: 1278.6671 Actual spec prec mz: 1278.667
Fragments matched per AA: 2.48 Proportion of top 20 peaks matched: 0.65
Intensity
1000000
750000
500000
250000
0
M/Z
500
1000
1500
2000
y1
y2
y3
y4
y6
y7
y10
y11
y13
y14
y15
y16
y17
y18
y19
y20
y22
y23
y24
B
Scatterplot of predicted elution time
Fitting R2: 0.716
Novel peptide residual Z score: 2.88
Number of peptides: 3314
Pred. hydrophobicity
Experimental scan number
0
20000
40000
60000
C
Distributions of residuals from best−fit line
of predicted RT vs Expt. scan number
Line: Z score of novel peptide
Z: 2.88
count
Z scores of residuals from best linear fit
−10
−5
0
5
10
